# Teenagers and Young Adults with Cochlear Implants: A Multidisciplinary Follow-Up Study Approach and Baseline Characteristics

**DOI:** 10.3390/audiolres15010016

**Published:** 2025-02-12

**Authors:** Ulrika Löfkvist, Malin Dahlby-Skoog, Anna Persson, Filip Asp, Luca Verrecchia, Susanne Gripenberg, Niki Karpeta, Martin Eklöf, Eva Karltorp

**Affiliations:** 1 Department of Clinical Science, Intervention and Technology, Karolinska Institute, 141 86 Stockholm, Sweden; malin.dahlby.skoog@ki.se (M.D.-S.); anna.persson.3@ki.se (A.P.); filip.asp@ki.se (F.A.); luca.verrecchia@ki.se (L.V.); eva.karltorp@regionstockholm.se (E.K.); 2 Department of Public Health and Caring Sciences, Uppsala University, 751 22 Uppsala, Sweden; 3 Medical Unit Ear, Nose, Throat, Hearing and Balance, Karolinska University Hospital, 141 86 Stockholm, Sweden; 4 Medical Unit Occupational Therapy and Physiotherapy, Karolinska University Hospital, 141 86 Stockholm, Sweden

**Keywords:** cochlear implants, adolescents, young adults, long-term outcomes, baseline characteristics, language understanding, study design

## Abstract

**Background/Objectives**: Early pediatric cochlear implantation positively impacts early language outcomes. The Teenager and Young Adults Cochlear Implant (TAYACI) study investigates the long-term outcomes of early implantation and factors influencing variability among cochlear implant (CI) users. This article outlines participants’ background, early language outcomes, and multidisciplinary study protocol. **Methods and Materials**: Individuals aged 12–22 received CIs before 30 months of age, followed-up at the same hearing implant center, and adhered to a standard school curriculum were invited to participate. Out of 109 eligible CI users, 50 participated; 46 agreed to undergo clinical assessments, while four completed questionnaires only. **Results**: The mean age at the first CI was 15.63 months (SD = 6.0). All but one communicated with spoken language(s). Participants attended mainstream schools and had highly educated parents. Over half (56%) had received Auditory Verbal Therapy in early childhood. Earlier implantation correlated with better language understanding one year post-CI. **Conclusions**: Earlier implantation was associated with better early language outcomes, with parental education level and early family-centered intervention likely contributing. Future sub-studies will investigate multidisciplinary long-term effects of pediatric cochlear implantation in adolescents.

## 1. Introduction

Cochlear implantation is a successful treatment for congenital and prelingual permanent severe-to-profound sensorineural hearing loss (SNHL). Most children with typical cognitive development who receive cochlear implants (CIs) at a young age, typically during infancy, are expected to acquire spoken language through listening and primarily follow a standard school curriculum [1,2]. Furthermore, long-term educational outcomes as well as quality of life are positively influenced by earlier age with a CI (<18 months) [3]. However, the population is heterogeneous and the hearing ability and spoken language development with CIs vary, despite early age at implantation. The understanding of which factors that affect this variation are only beginning to emerge [4]. There is a need to not only understand the outcomes in early childhood, but also the complexity of long-term CI intervention outcomes. This would preferably be studied in population-based cohorts in order to adopt more specific and tailor-made guidelines and recommendations in the hearing care system [5].

Older school-aged children and adolescents are in transitional phases of development, moving from childhood through adolescence into adulthood. Little is known about how CI users navigate these developmental stages while simultaneously continuing to adapt their listening abilities with CIs, in complex listening and learning environments with varying amounts of support. Only a few large-scale studies have investigated long-term cognitive skills in children with CIs [1,2,6]. For school-aged children, previous research findings show that early age at first CI affects speech recognition and speech, and may indirectly also affect cognitive skills like reading and language abilities [2,4]. Long-term outcomes after pediatric CIs are particularly sparse related to finer linguistic competence, general well-being, and listening skills in more complex listening conditions. In addition, it is also unclear how the chronic electrical stimulation in the inner ear and the CI procedure itself affects vestibular function over time and manifests in teenagers and young adults who received CIs at an early age.

In our group’s cross-sectional and longitudinal clinical studies, we demonstrated that age at first CI is a critical factor for early spoken language development but does not significantly influence speech recognition [7,8,9]. However, some of these findings were based on retrospective analyses, lacked control groups, or included children who were too young to participate in certain assessments. Limited clinically based genetic screening was conducted, and assessment of basic auditory skills was not always performed. In addition, we have previously not included multilingual participants in our studies, although around 30% of all children who receive CIs at the Hearing Implant Center (HIC), Karolinska University Hospital, use several languages for communication [10]. Multilingual cohorts are generally understudied in the literature, which is problematic from many perspectives, especially in relation to health-care equity [11,12]. Another demographic factor that is known to influence early language development is the socioeconomic level of the family [13], which may also affect long-term outcomes in teenagers and young adults.

Approximately 0.2% of all newborns in Sweden are born with hearing loss, with 0.04% of them having a severe-to-profound bilateral SNHL [14]. The population of individuals with prelingual deafness who use CIs is highly heterogeneous, partly due to variations in the duration of auditory deprivation or reduced hearing experience before undergoing CI surgery [15]. Although the Universal Newborn Hearing Screening (UNHS) system was not fully implemented across all regions of Sweden until 2008, its adoption has since led to a gradual reduction in the age at which most children with congenital or prelingually acquired deafness receive their first CI.

During the time period 2018–2023, 39.3% (*n* = 328) of all Swedish children with prelingual deafness received their first CI before 12 months of age [16]. Previously, in the time period of 2000–2011, which are the birth years of the participants in the present follow-up study, the mean age at first CI was somewhat older. During this time period, only 14.9% (*n* = 424) of all Swedish infants received a CI before 12 months [16]. Other hearing care factors have also changed in the Swedish system gradually. Today, children receive simultaneous bilateral CIs, or develop listening and spoken language through bimodal hearing, using a combination of a unilateral CI and hearing aid. Furthermore, Family-Centered Early Intervention (FCEI) options, like Auditory Verbal Therapy (AVT) [17], have been introduced on a national basis since around 2005. These advancements, particularly the introduction of early preventive interventions, have resulted in better opportunities for children born deaf to develop listening skills and spoken language on par with their chronological-aged peers with typical hearing (TH) [9].

There is solid support in previous research that early implantation has positive effects on linguistic and cognitive development [1,18,19]. Albeit shown by our previous work that implantation before nine months is ideal [9], the large variability in the linguistic performance of children growing up with CI cannot fully be explained by age at implant [4]. For example, differences in executive functions could be related to the variability in linguistic performance. The development of executive functions is closely linked to linguistic development [20,21], and studies have shown differences in the relationship between executive functions and language in individuals with CIs compared to peers with typical hearing [22,23]. When it comes to higher linguistics skills, such as understanding metaphors, research in individuals with CIs is still scarce. A handful of studies has found difficulties with metaphor comprehension in individuals with CIs [24,25,26]. There is, however, further need to explore both metaphor comprehension and executive functions, and their relationship, in early implanted individuals.

Hearing, a fundamental part of verbal communication, shows large outcome variability after cochlear implantation in children [27]. The hallmark of hearing is the recognition of speech. Studies show conflicting results regarding the impact of early implantation on speech recognition in quiet conditions [1,7,28,29], whereas it is widely accepted that children with CIs are more susceptible to noisy conditions than children with typical hearing [6,7]. Little is known, however, about long-term performance in children of an early age at CI surgery, specifically in more naturally occurring and complex listening environments, in which they spend much time communicating and learning. An example of a complex listening environment is a condition in which a listener wants to understand speech in the presence of masked speech. We are aware of a few studies measuring performance for such conditions in children with CIs at 6 and 9 years of age [30,31] but not in teenagers and young adults with early implantation. Misurelli et al. [30,31] observed large variability in performance for children using implants in conditions with interfering speech. The source of this variability is poorly understood. Another example of an important auditory ability that may aid daily life communication is sound localization. We and others have shown that horizontal sound localization accuracy is better with bilateral than with unilateral CIs [7,32]. Sound localization seems to develop gradually after bilateral input is provided despite relatively late and/or sequential implantation [33], but simultaneous implantation allows higher accuracy in children aged 7–10 years [34]. It is unknown if the benefit of simultaneous implantation is maintained into adolescence and early adulthood, a question we address in this study.

The inner ear balances (vestibular) organ functions as a complex inertial sensor which detects the head movements/positions and adjusts the muscle activity in the trunk, limbs, neck, and eyes for an optimal gaze stabilization and body posture during movements and stance. The vestibular organ consists of two otolith organs (sacculus and utriculus, activated by linear accelerations) and three semicircular canals (activated by angular accelerations). Accumulated clinical evidence has revealed a vestibular deficit in children who are deaf and hard of hearing (DHoH) with high prevalence [35], and a potential detrimental effect of cochlear implantation on vestibular function [36,37]. Moreover, the presence of a vestibular impairment, especially in the form of a bilateral vestibular loss, has been shown to alter the motor development of young children [38], and the motor proficiency in school-aged/teenaged CI recipients [39,40]. Impaired gaze stabilization, reading difficulties [41], acquisition of protective fall reactions [42], and cognitive abilities [43] have also been associated with vestibular failure in developmental ages. The impact of vestibular impairment on the motor abilities, hearing, speech, and cognitive function of early CI recipients grown up to school/teenage age is still debated and in need of further investigation [44,45,46].

Another factor that contributes to the heterogeneity in individuals with CIs is etiology, or the cause of SNHL or deafness. Around 30–40% of all children who are deaf or hard of hearing have additional diagnoses that may affect their outcomes [47]. Knowledge of the cause of a child’s deafness or SNHL is crucial, both for the individual and also for health-care professionals and caregivers so they can support children and teenagers who are DHoH in optimal and efficient ways. The most common causes of deafness and SNHL are due to genetic mutations in genes that are important for hearing pathway development and auditory functioning. Gene mutations are believed to be the cause of at least 50% of SNHL and deafness in children [16]. Thirty percent of the genetic causes of SNHL are syndromic, including examples such as Jervell and Lange-Nielsen syndrome, Waardenburg syndrome, Pendred syndrome, and Usher syndrome. Common causes of congenital and acquired SNHL in children include congenital infections, such as rubella or congenital cytomegalovirus (cCMV) infection, which alone accounts for approximately 5–20% of cases [48]. However, in the Western world, rubella is nowadays rare due to vaccination programs, but cCMV infection is still very common [49]. Meningitis is now rare in Sweden due to the introduction of the pneumococcal vaccine into the national childhood vaccination program in 2009 [50]. Most newly identified Swedish children with hearing loss are nowadays tested both genetically for mutations known to cause hearing loss (GJB2) and for congenital CMV infection (not screened at birth). Hence, the cause of a child’s deafness or SNHL is known for more children, but still not for all individuals. Possible reasons why not all children have a conclusive etiology include the gradual evolution of clinical diagnosis practices over time, which have varied across different counties in the country. Additionally, all genetic mutations causing hearing loss have not been identified, and the cost for extensive genetic testing in clinical settings may not be feasible for everyone. Most children born with cCMV infection experience progressive SNHL, which is currently not detected at birth during the universal newborn screening. As a result, many children with cCMV infection undergo a delayed hearing and cCMV diagnostic process. In Sweden, a retrospective CMV diagnosis can be made using a dried blood test (PKU-test) that is conducted at birth on all children and stored for potential future analysis. Identifying the cause of SNHL can provide a clearer prognosis for the child and ensure that appropriate support is offered based on the diagnosis.

Mental health and health-related quality of life (HRQoL) cover a wide and comprehensive outcome area. In this project, these areas are especially related to everyday listening experiences with CIs in different environments and situations, and the participants’ well-being. The heterogeneity in outcomes found in this group may be related to comorbid conditions due to etiological factors and may not only influence language, hearing, and social skills, but also mental health. Previous studies have mainly been based on questionnaires, where parents of children with CIs have reported on their behalf. Furthermore, few interview studies of adolescents with CIs have been conducted, and only in small groups with a large variation in age at first CI [51,52,53]. This may be due to the fact that it is not until recently that individuals that were implanted at an early age have become teenagers and young adults and are able to speak for themselves.

The risk of depression and other mental health-related problems increases dramatically during adolescence, and therefore the prevention of depression during this period is especially prioritized for all adolescents [54,55]. Previous studies have reported that children with CIs have more problems with mental health than peers with typical hearing [56]. As an example, children and teenagers with CIs have previously been shown to have more depressive symptoms than age-matched peers with typical hearing [57]. One of the traits known to have a protective effect against depression is higher levels of self-efficacy [58]. This factor has been investigated in parents of young children who are DHoH [59,60] but less so in the target group of the present study. Prospective multidisciplinary evaluation of higher-level cognitive skills in relation to listening skills and HRQoL is highly motivated. By conducting such research in a larger group of teenagers and young adults who have grown up using CIs from early childhood, and matching them with controls with typical hearing, we will gain new valuable insight. This new knowledge may contribute to understanding the specific clinical needs of subgroups and individuals with CIs.

This far, there are only a few previous longitudinal, multicenter, and population-based studies that have examined children who are DHoH. These studies provide valuable insights into the population by addressing the sample size limitations often encountered in single-center studies. Such research has primarily been conducted in Anglo-Saxon countries like Australia and the USA. One example is the population-based Longitudinal Outcomes of Children with Hearing Impairment (LOCHI) study from Australia which included 460 children with different types of hearing loss and deafness [61], but to our best knowledge they have not yet investigated the outcomes of teenagers. At five years of age, children in the LOCHI study with severe or profound SNHL who received CIs before 12 months of age had significantly higher language scores than those who received CIs at an older age [62]. In the US, the Childhood Development After Cochlear Implantation (CDaCI) study, outcomes of children with cochlear implants were compared to age-matched children with typical hearing [63]. Another US study is the Outcomes of Children with Hearing Loss (OCHL) study, examining different factors influencing language and auditory outcomes in young children with permanent, bilateral, and mild to severe-to-profound SNHL [64]. The findings of these studies are highly valuable but may not be fully representative of other regions and countries worldwide.

The aim of this article is to describe the Teenagers and Young Adults with Cochlear Implants (TAYACI) study, specifically focusing on its design and approach across five key study domains: language and cognition; hearing and listening; balance; etiology; and mental health and HRQoL. It also presents background characteristics of participants with CIs and their families and examines early hearing and language development in relation to the age at first CI. The TAYACI study is a national cohort study, with participants followed-up at the same CI center serving half of Sweden’s population.

The study addresses two primary research questions:

Q1: How do teenagers and young adults who received their first CI before 30 months of age perform long-term in language, cognition, hearing, balance, self-efficacy, and HRQoL, compared to age-matched controls with typical hearing?

Q2: How do adolescents and young adults with CIs perceive their listening and communication experiences in different everyday life (e.g., school, work, leisure) compared to controls with typical hearing?

The hypotheses that will be tested across the five sub-studies of the TAYACI study are as follows:

Q1: Age at first CI impacts long-term cognition, language, hearing, and HRQoL.

Q1: Hearing outcomes are related to modifiable clinical parameters.

Q1: Lower socioeconomic status correlates with poorer long-term outcome (cognition, language, and HRQoL).

Q1: Etiological factors influence balance, hearing, language, cognition, and HRQoL.

Q2: Self-perceived HRQoL is positively affected by age-equivalent language skills and better hearing.

Q2: Overall HRQoL in adolescents and young adults with CIs is comparable to that of age-matched controls with typical hearing.

## 2. Material and Methods

### 2.1. Study Design

A cross-sectional study utilizing retrospective and follow-up data collection with a multidisciplinary approach, incorporating both quantitative and qualitative methods.

### 2.2. Brief Prospective Project Overview

The ongoing research program consists of five sub-studies (Sub-Study I–V) in the same cohort of individuals with CIs. Inclusion criteria: adolescents who had received their 1st CI before the age of 30 months and were followed-up at the HIC, Karolinska University Hospital, aged 12–22 years, and who follow or have followed normal school curriculum were asked to take part in Sub-Studies I-V during the period 2022–2023. Participants were invited to the clinic for an extended research visit at the time point of their regular clinical appointment. The multidisciplinary research team consisted of two principal investigators, a speech–Language Pathologist (Sub-Study I), a clinical audiologist, an engineer and radiologist (Sub-Study II), two balance specialists (medical doctors) and a physiotherapist specialist (Sub-Study III), a medical doctor specialized in audiology (Sub-Study IV), and a teacher of the deaf (Sub-Study V). Saliva samples were taken at HIC for those with unknown cause of deafness and who specifically volunteered for this investigation (Sub-Study IV). High resolution photon-counting computer tomography was performed to estimate electrode insertion depth and scalar location. Information from previous medical records and the clinical software (Maestro, version 11.0.2, for Med-El and Custom Sound 7.0, for Cochlear) and for cochlear implant fitting were collected and used as background material and as predictors for hearing and language outcomes, respectively. The adolescents filled out questionnaires either before the follow-up visit or during the test occasion, with support provided if needed. Additionally, they were invited to participate in semi-structured focus group interviews or individual interviews scheduled after the follow-up visit at HIC. Caregivers of participants under 18 years of age also filled out some questionnaires.

### 2.3. Participants

Initially, we recruited a clinical cohort of participants with CIs at the HIC, Karolinska University Hospital. Thereafter, we recruited controls with typical hearing who were matched for age. Control groups were recruited for each sub-study (I–V), a mainly overlapping cohort. Descriptive information about controls will be shared in later publications.

**Figure 1:**
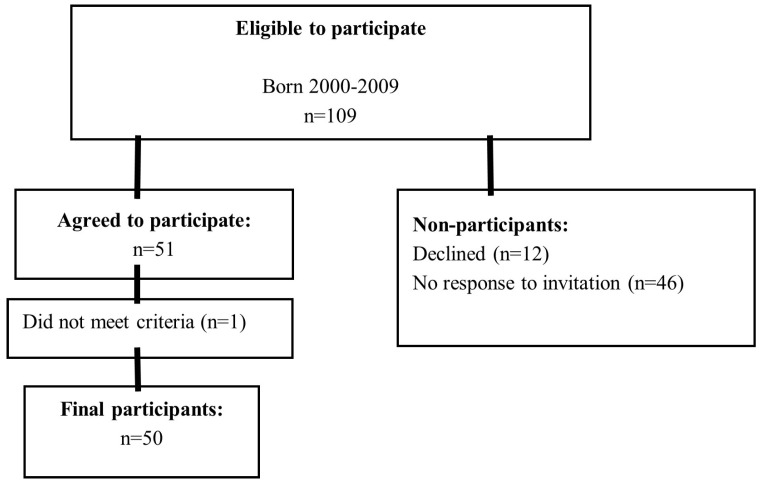
Flowchart illustrating the study inclusion process.

A total of 135 individuals met the criteria of age at implantation <30 months and were aged 12–22 years in the clinical database. Twenty-six individuals were excluded due to non-fulfillment of inclusion criteria (other curriculum). Invitations were sent to 109 individuals who were followed-up at Karolinska University Hospital and met the study criteria. Twelve declined to participate, 51 accepted the invitation and the remaining 46 did not respond. One participant was incorrectly invited from start (did not meet the inclusion criteria of age at CI 1) and was therefore excluded from the study (Figure 1). The final number of participants was 50 (27 females and 23 males), aged 12–22 years (see Table 1 in the results section). The majority used spoken Swedish as their primary communication mode (*n* = 42), three participants used at least two spoken languages in their daily communication. Another four participants used spoken Swedish with sign support (*n* = 4) and one participant primarily used sign language and some spoken Swedish (*n* = 1). For further demographic background information and hearing characteristics see Table 1 and Table 2.

#### 2.3.1. Characteristics of Participants with CI and Their Families

Descriptive background for the group (*n* = 50) is presented in Table 1 and Table 2, focusing on hearing-related factors, etiology status at the time of recruitment, and family characteristics during the follow-up data collection.

The educational level of the participants’ parents may be somewhat higher than expected in the population (Table 1). According to population-based data, the proportion of highly educated individuals has increased significantly since 2000, rising from 16% to 30%, while the number of those with lower education levels has declined, except among immigrant groups [65]. In 2021, it was estimated that 52% of women and 39% of men aged 25–64 had completed post-secondary education [65]. Educational attainment is notably higher in larger cities, and among individuals aged 45 and older, suggesting that Swedish adults commonly continue their university education later in life.

#### 2.3.2. Early Follow-Up Procedures and Habilitation Actions After First CI

The participants with CIs come from different parts of Sweden (29 from the capital area and 21 from other regions). They have been followed-up by the same multidisciplinary cochlear implant team at the HIC, Karolinska University Hospital, on a regular basis with a standardized protocol. An educational audiologist has investigated the speech recognition, a speech–language pathologist has assessed spoken language abilities, and an engineer has controlled and mapped their CI(s) at the same occasions. Furthermore, they have sometimes met audiologists (MD) to discuss medical and etiological issues and more rarely a social worker to discuss questions related to insurance and support actions in the local hearing care after surgery, etc. After the initial investigation, surgery, and first fitting, post-operative evaluations were performed 6 months after surgery, and then every 6 months until the children were four years. Thereafter, they have been assessed at the same center on an annual basis until 17 years of age. All participants except one have received some sort of Family-Centered Early Intervention (FCEI) in their local hearing care team. Twenty-eight families had received Auditory Verbal Therapy (AVT) on a regular basis (at least two sessions per month during the first year after initial CI-fitting), while 22 families had received unspecified FCEI services, with a more auditory oral or total communication approach, and with unclear frequency and intensity (see Table 2). There was a clear dominance of AVT provided in the capital area (*n* = 22), and only six families who received AVT in other regions.

### 2.4. Descriptions of Material and Procedures Used in Sub-Studies (I–V)

Background information (demographics) and academic level: Background information (family background, family socioeconomic status level, communication mode, school setting, reading, and screen use habits) and questions about national school test results: Swedish, Mathematics and English at 6th and 9th school year levels and in high-school) (www.skolverket.se, accessed on 1 December 2024). Information about early hearing background characteristics like age at CI surgery, and language outcome results after 1st CI have been collected from medical records at the HIC, Karolinska University Hospital, with consent from participants. This article presents background information from medical records available at the time of data collection, baseline questionnaires completed by participants, and clinical data related to early outcomes. For an overview of the entire test battery and the specific instruments used in Sub-Study I–III and V, see Supplementary Materials (S1).

#### 2.4.1. Sub-Study I: Language and Cognition

During one test session, participants were assessed on language and cognition, including executive functions, reading, lexical–semantic abilities, and metaphor comprehension (S1). Since there is no standardized test assessing metaphor comprehension in Swedish, a new task was devised for this study, based on previous work by Kalandadze and colleagues [66]. The metaphor task includes multiple-choice responses as well as a verbal explanation. Responses will be analyzed both quantitatively and qualitatively. Metaphor comprehension and executive functions task performance will be analyzed separately, in relation to each other, as well as in relation to lexical–semantic abilities. The total test time for the language and cognition tasks was three hours, which included two ten-minute breaks for each participant. Participants and their parents also filled out one or two questionnaires during their visit, which are included with the additional documents and questionnaires used in Sub-Study V (S1).

#### 2.4.2. Sub-Study II: Hearing and Listening

The participants’ hearing was tested in a 2.5 h visit including short breaks between the tests (S1). Participants were always tested in bilateral listening conditions, e.g., bilateral CIs or bimodal listening, except for the measurement of aided sound-field thresholds which were recorded for left, right, and bilateral listening conditions.

Hearing thresholds

Aided sound-field hearing thresholds were recorded using frequency-modulated tones with center frequencies at 0.125, 0.25, 0.5, 1, 2, 3, 4, 6, and 8 kHz for left, right, and bilateral listening conditions. Measurements were performed in a quasi-free sound field in an audiometric test room (4.1 m × 3.3 m × 2.1 m) with a low ambient sound level (mean = 25 dB (A) obtained during 15 sec measurement) and short reverberation time (T30 = 0.11 s at 500 Hz), as recorded with a B&K 2238 Mediator and B&K 2260 Investigator (Brüel & Kjær, Nærum, Denmark), respectively. Participants were seated at a distance of 1 m from the loudspeaker presenting the stimuli at frontal incidence. Thresholds were determined according to ISO 8253-2:2009.

Recognition of words in quiet

Speech recognition was assessed in a quasi-free sound field in an audiometric test room (same as above used for the assessment of hearing thresholds) using a standardized and validated Swedish clinical speech audiometry test [67]. The test material consisted of phonemically balanced lists of 50 monosyllabic words. Two lists at 65 dB SPL and 50 dB SPL, respectively, were presented. Participants were seated 1 m away from a loudspeaker, with the words presented at frontal incidence. Participants were required to repeat a word after hearing it, and the response was determined as correct or incorrect by an audiologist who was sitting outside the audiometric test room listening through a feedback system. Responses had to be grammatically correct to be scored as correct. The final scores for both presentation levels were the proportions of correctly recognized words.

Recognition of sentences in masking speech

An adaptive psychoacoustic task was used to estimate the 40% recognition threshold for target speech in co-located and spatially and symmetrically separated masking speech [68,69]. Target sentences were the Swedish Hagerman sentences (female voice, [70]), whereas the masking speech consisted of four noncorrelated speech signals (one male voice reading out of a novel). Measurements were performed in sound field in a double-walled sound booth (4.0 m × 2.6 m × 2.1 m) with a mean ambient sound level = 20 dB (A) obtained during 15 sec measurement and reverberation time T30 = 0.09 s at 0.5 kHz, as recorded with a B&K 2238 Mediator and a B&K 2260 Investigator (Brüel & Kjær, Nærum, Denmark). Participants were seated 1.8 m from the loudspeaker presenting the target signal, positioned at 0° azimuth. Four loudspeakers were placed in the corners of the room (±30° azimuth and ±150° azimuth). The target speech was the Hagerman sentences [70]. Each sentence consisted of five words that formed a grammatically correct sentence with low semantic predictability in a fixed syntax (e.g., “Peter höll nio nya lådor”, in translation: “Peter held nine new boxes”). Twelve lists (and one training list), each containing 10 sentences, were used. The interferers comprised four noncorrelated recordings of a single male talker reading a novel. The interferers were presented either from the four corner-placed loudspeakers or co-located with the target signal (0° azimuth) at a fixed overall level of 63 dB SPL (12 min recording time) [68].

Participants were instructed to face the frontal loudspeaker during the entire test but not informed that the target signal originated from 0° or about the different spatial configurations (separated and co-located) to avoid influencing the test [71]. They were asked to repeat the words of one training list (always the same list) and two target lists, and their oral responses were recorded by an experimenter outside the test room. The experimenter listened to the target signal and the participant’s responses through a feedback system and scored the responses after each sentence. Guessing was encouraged, and no feedback was provided. Words had to be repeated grammatically correctly to be scored as correct. The training started at a SNR of +10 dB. For the following training sentences, the target speech level decreased up to three times in 5 dB steps, then up to three times in 3 dB steps, and then in 2 dB steps until the number of correct words in a sentence was ≤2. Upon completion of the training, the level adjustment of the target speech was +2 dB for zero correctly identified words, +1 dB for one correctly identified word, 0 dB for two correctly identified words, −1 dB for three correctly identified words, −2 dB for four correctly identified words, and −3 dB for five correctly identified words, aiming at a threshold of 40% words correct. The 40% threshold and the adaptive scheme for level adjustment were based on computer simulations and analysis of the maximum steepness of the psychometric function [70,72,73]. The SRT was defined as the mean of the SNRs for the last 10 presented sentences [73,74].

Horizontal sound localization accuracy

Horizontal sound localization accuracy was measured in the frontal horizontal plane (12 sound-sources evenly distributed across ±55 degrees azimuth; 10 degrees between target sound-sources) using an objective, rapid, and reliable technique utilizing eye-gaze responses [75]. Measurements were performed in a quasi-free sound field in an audiometric test room (4.1 m × 3.3 m × 2.1 m). The approximate distance from the head of the participant to the loudspeakers was 1.2 m. The loudspeakers were vertically adjusted to accommodate different heights of the sitting participants and to achieve loudspeaker positions at approximate ear level. Seven-inch video-displays were mounted below each loudspeaker, resulting in twelve loudspeaker/display-pairs (LD pairs). The video-displays were visible, while the loudspeakers and the loudspeaker-stand were covered in black cloth. An eye-tracking system (Smart Eye Pro, Smart Eye AB, Gothenburg, Sweden) was used to record the gaze of the participants in relation to the LD pairs (see [75] for details). The coordinates of the video-displays and loudspeakers were defined in three dimensions in the eye-tracking system, resulting in 12 Areas of Interest (AOI) each of which had a width of 0.17 m and height of 0.55 m, and in total they constituted a continuous array of AOIs in a 3D model, corresponding to the physical LD pairs.

To assess the contribution of different spatial cues to sound localization, four different stimuli were used [76]. Two stimuli were broadband and thus made interaural time and level difference cues available. These stimuli had long-term frequency spectra similar to the spectrum of a female voice. One of these two stimuli was a musical melody which had naturally occurring amplitude modulations, thus minimizing monaural level cues. The other broadband stimulus was a stationary noise, increasing the availability of monaural level cues compared with the musical melody. The rationale for using these two stimuli was to allow a comparison between conditions with and without distinct monaural level cues while ITD and ILD cues were available. To assess the contribution of ILD and ITD cues to localization accuracy separately, localization accuracy was also measured using two octave-filtered noises with center frequencies at 0.5 and 4 kHz, providing interaural time and level cues, respectively. The 4 kHz stimulus also allowed access to monaural level cues, whereas the 0.5 kHz stimulus minimized monaural cues. All four stimuli were presented at 63 dB SPL.

Immediately before each test session, a calibration of the participants’ gaze relative to the visual displays was performed [75]. The stimulus was presented continuously and started from the LD pair at −5°. After an average time interval of 7 s, the visual stimulus was stopped and the sound was instantaneously shifted to a randomized loudspeaker. The visual stimulus was reintroduced after 1.6 s to allow for sustained acquisition of the gaze toward the video screens. During the 1.6 s sound-only period, the subjects were guided by audition only as to where the active sound source was located. The auditory and visual shifts were repeated 24 times at random with the constraint that no loudspeaker/display was used a second time before each of the 12 loudspeaker/display pairs had been used. Participants’ pupil positions relative to the loudspeaker/display pairs were sampled at 20 Hz. The resulting gaze/AOI intersections were derived from the output of the eye tracker and stored as a function of time. The perceived auditory azimuth was defined as the median of the final 10 gaze/AOI intersection samples obtained during the 1.6 sec sound-only period, that is, a 500 msec sampling period.

Interaural level and time differences

A psychoacoustic test of the just noticeable difference in the interaural level and time difference in a 250 Hz pure tone was administered. Headphones (HDA200, Sennheiser) were used together with the PsychoPy test suite (University of Nottingham) [77] in an adaptive 2-up-1-down 1I-2A forced choice paradigm [78].

Assessment of programming levels and objective and behaviorally assessed thresholds

The participants went through a fitting session before the language and hearing tests to ensure full technical functionality. No changes in programming levels were introduced at this occasion. The electrical comfort and threshold levels of the participants’ clinical maps were collected, and the actual electrical threshold was assessed behaviorally by means of the modified Hughson–Westlake method at three positions along the electrode array. Additionally, electrically evoked compound action potentials were measured when possible.

Spectral discrimination

A psychoacoustic test of spectral discrimination previously used in adults [79] was used to assess the peak–valley ratio (PVR) in dB at 4 and 8 frequency channels. Briefly, the sound was created by alternating the amplitude in frequency bands of a pink noise. Each sound was created in two versions where the first half of the sound had odd bands attenuated and the second half of the sound had even bands attenuated to ensure equal loudness. Each sound pair was created in 10 versions with unique frozen pink noises to make sure the subject would not be able to learn to recognize the character of the noise. One of the 10 versions were chosen randomly for each iteration. The procedure followed an adaptive staircase 2-down-1-up 3I3A forced choice paradigm, preceded by a training session.

Photon-counting computer tomography

Photon-counting computer tomography was performed in a subset of participants to reconstruct inner ear anatomy with high resolution, determine scalar location for CI electrodes, and quantify interaural asymmetries of angular insertion depth (AID).

#### 2.4.3. Sub-Study III: Balance

The balance function has been studied at different levels.

Vestibular function: The vestibular responses (vestibular reflexes) were investigated with two modern clinical tests: the video head impulse test (vHIT) and the VEMP (vestibular evoked myogenic potentials). vHIT is the recording of eye velocity at the peak of head velocity during passive head jerk rotations on the planes of semicircular canals [80]. The main measure is the gain of eye velocity on head velocity which is directly correlated to the residual function in the semicircular canals. By vHIT, it is possible to obtain separately the VOR gain of each of the three semicircular canals (anterior, lateral, and posterior) for the two sides. The VEMP is the recording of short latency muscle responses at cervical muscles (cervical VEMP, cVEMP) and eye muscles (ocular VEMP, oVEMP) in response to vestibular activation by impulsive air conducted sounds (AC) or skull vibrations (bone conduction, BC) [81]. cVEMP is considered a test of sacculus function and oVEMP of utricular function. By vHIT and VEMP, it is possible to map in detail the function of the different parts of the vestibular organ and the integrity of the vestibular pathway.

Balance tests: An assessment of motor function, balance, and gait, blinded to vestibular testing, was conducted by a physiotherapist using the subtest of balance from the Bruininks–Oseretsky Test of Motor Proficiency (BOT-2), the sections Reactive Postural Responses and Sensory Orientation from Kids-Balance Evaluation System Test (Kids-BESTest) [82], walking 10 m with and without head turns [83], and the head impulse test (HIT). In addition, self-reported physical activity and experience of balance were measured with a self-composed questionnaire and SGPALS [84]. The aim of this testing was to ascertain possible balance and motor deviations at common clinical testing in CI recipients and to find possible associations between balance alterations and vestibular impairment. Motor pattern recording: A quantitative analysis of balance and motor functioning was conducted with inertial sensors. All participants wore a total of 7 wireless inertial sensors attached to different body parts of the kind APDM during the balance tests [85]. This was performed to obtain a parametric analysis of their motor pattern. Each sensor could measure the intensity, direction, and duration of the seven body segment movements during testing. The data obtained were analyzed with machine learning and AI analysis. For the technical specifications, the protocol, and the parameters used in the studies, see Supplementary Materials (S1).

#### 2.4.4. Sub-Study IV: Etiology

The cause of deafness or sensorineural hearing loss (SNHL) was identified in 74% (*n* = 37) of the cohort at the time of the follow-up study (see Table 1). This relatively high proportion of known diagnoses can be attributed to the early development of a clinical protocol for etiological investigation at the HIC, Karolinska University Hospital, implemented at the time participants underwent CI surgery. The protocol included targeted screening for cCMV infection and genetic testing for GJB2 mutations (connexin 26), two of the most common causes of deafness and SNHL. Additionally, the HIC collaborated with referring local audiological clinics and conducted parental interviews during the cochlear implant investigation. These interviews gathered information on the child’s general health and any family history of hearing loss, facilitating the identification of hereditary causes. The thirteen participants with an unknown diagnosis were offered a clinical diagnostic investigation, including a genetic test panel (saliva sample), and all agreed to participate. Through this procedure, we hope to determine the etiology for the majority of participants with CIs, facilitating subgroup analyses of different etiologies in relation to various outcomes in Sub-Studies I-V. This effort will contribute new insights to the literature on how balance, hearing, language performance, mental health, and HRQoL may be influenced by different etiological factors.

#### 2.4.5. Sub-Study V: Mental Health and Health-Related Quality of Life

To investigate mental health, everyday life experiences, and HRQoL, a mixed-methods design was employed, integrating both quantitative (questionnaires) and qualitative (focus groups, individual semi-structured interviews, and text analysis) approaches (S1). Music perception and appreciation have been shown to be related to the quality of life in individuals with CIs [86,87] and will be explored in greater depth in our project. As part of the HRQoL assessment, participants are asked about their music habits and appreciation through both questionnaire and interviews, aiming to provide a deeper understanding of music perception in CI users who were implanted at a young age. Participants received questionnaires along with a document containing demographic questions on the first day of testing, with responses collected on the second day. This process allowed participants to provide feedback on the instruments and seek clarification about any questions that arose while completing the questionnaires.

All participants were informed that they would receive a separate invitation to join a focus group shortly after their on-site assessments. For those unable or unwilling to participate in focus groups, individual semi-structured interviews were offered as an alternative. These interviews were analyzed using content analysis [88].

### 2.5. Statistics

Statistical analysis was performed using JASP (version 0.19.0) and RStudio (version 2022.07.2 + 576) and SPSS (version 28). The baseline characteristics, socioeconomic status, and retrospective outcomes of the study group are summarized using descriptive statistics at the group level. Shapiro–Wilks tests showed that the RDLS-III results were not normally distributed. Based on this, Spearman’s rank correlation analyses were performed to examine the relationship between age at 1st CI and language understanding after one year with the CI. In future manuscripts, the selection of appropriate statistical methods will depend on the sample sizes of the control groups and the data distribution in each Sub-Study (I–V). These methods may include tests for group differences, correlations with additional factors, and, where feasible, regression analyses. A pair-wise deletion approach to missing data was used for calculations for the current paper. In future manuscripts, approaches to missing data will be determined by sample size and data distribution.

## 3. Results

### 3.1. Representativeness of Study Sample

**Figure 2:**
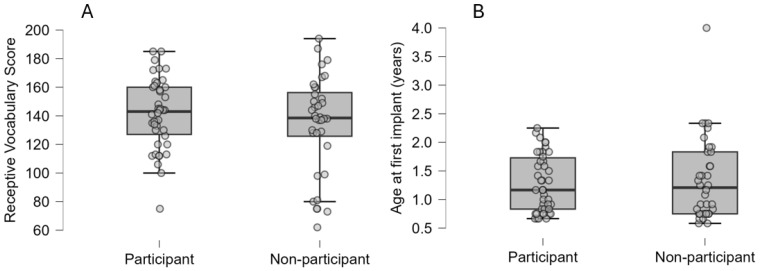
Comparison of receptive vocabulary outcomes at approximately 10 years of age and age at implantation between participants and non-participants. (**A**) = receptive vocabulary scores for participants and non-participants, (**B**) = age at first implant (years) for participants and non-participants.

To assess whether the participants were representative of the broader population, we analyzed previously collected clinical data on receptive vocabulary for those who agreed to participate (*n* = 50) and those who declined or did not respond to the invitation (*n* = 58). Data for the Peabody Picture Vocabulary Test, PPVT (3 and 4) [89,90], were available for 42 of 50 (84%) of the participants at a mean age of 10.0 years (SD = 1.0 years) and 36 of the 58 (62%) non-participants at a mean age of 10.1 years (SD = 0.9 years). The analysis revealed no differences in receptive vocabulary outcomes (*p* = 0.30, independent samples *t*-test) (Figure 2A), or age at implantation (*p* = 0.65, independent samples *t*-test) (Figure 2B) between participants and non-participants.

### 3.2. Language Understanding After One Year with First CI

**Figure 3:**
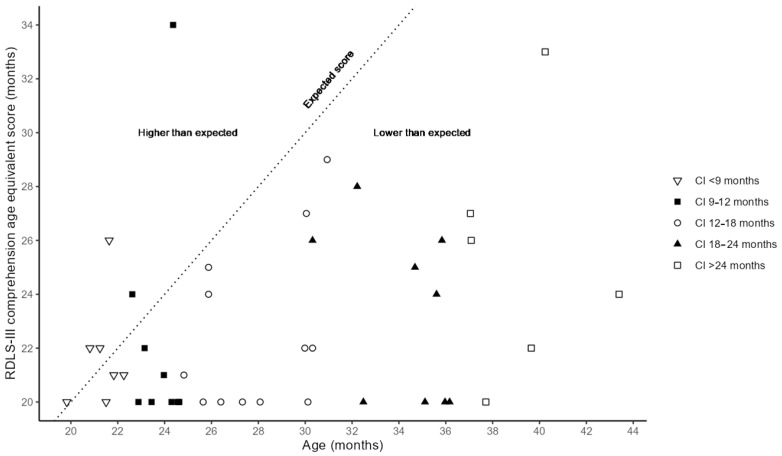
Language understanding after one year with 1st CI. RDLS-III age equivalent comprehension scores (mean = 22.9 months, range = 20–34 months*) at one year post 1st CI (*n* = 43, mean age = 28.9 months, range = 19.9–43.4 months, mean age at 1st CI = 15.8 months, range = 7.3–29.3 months). Five groups were created based on age at 1st CI; CI <9 months (*n* = 7, mean age = 21.3 months, mean age at 1st CI = 8.3 months), CI 9–12 months (*n* = 9, mean age = 23.8 months, mean age at 1st CI = 10.7 months), CI 12–18 months (*n* = 12, mean age = 27.9 months, mean age at 1st CI = 14.9 months), CI 18–24 months (*n* = 9, mean age = 34.3 months, mean age at 1st CI = 21 months), and CI > 24 months (*n* = 6, mean age =39.2 months, mean age at 1st CI = 26 months). Dotted line illustrates expected score for age, scores above the line are higher than expected and scores below the line are lower than expected. Note: RDLS-III = Reynell Developmental Language Scales III, * < 21 months is the lowest possible score of RDLS-III, 20 months was used for this score.

In our previous retrospective study, we found that earlier ages at first CI were associated with better language understanding in the early years [9]. In this study, we investigated whether the same pattern holds for the current cohort. Language development is regularly assessed at the HIC, Karolinska University Hospital, to evaluate outcomes after cochlear implantation. We present data on language understanding using the third version of the Reynell Developmental Language Scales (RDLS-III) 12 months post implantation, a tool that evaluates receptive and expressive language abilities in children aged 0–7 years [91] (see Figure 3). A validated Swedish version of the comprehension section was used, with results comparable to English norms for children aged 2.5–3.4 years. Due to the narrower age range in the Swedish study, we used validated English data for comparisons. RDLS-III scores 12 months post implant were available for 43 participants (see Figure 3). A Spearman’s rank correlation was computed (*n* = 42, excluding one participant who was younger than the lowest possible RDLS-III score). No correlation was found between chronological age and RDLS-III age equivalent score (r = 0.214, *p* = 0.173, 95% CI [−0.11, 0.49]) or between age at first CI and RDLS-III age equivalent score (r = 0.238, *p* = 0.128, 95% CI [−0.08, 0.51]). However, a positive correlation was found between age at first CI and the differences between age and the RDLS-III age equivalent score (mean = 6; range = −9.6 to 19.4) (r = 0.851 *p* < 0.001, 95% CI [0.73, 0.92]). This suggests that in our cohort at 12 months post implantation, a lower age at first CI was associated with test performance closer to age-expected levels.

## 4. Discussion

The main motivation for conducting the inter-professional TAYACI study is the limited in-depth knowledge in the international literature regarding long-term effects of age at cochlear implantation in deaf adolescents and young adults, who were implanted in early childhood, especially outside Anglo-Saxon areas and countries like the US and Australia. This study examines specific aspects, including executive functioning, linguistic abilities including metaphor comprehension, hearing and balance outcomes, self-perceived HRQoL, mental health, and self-efficacy among individuals with CIs from different parts of the country. Notably, 51% of all eligible CI users (*n* = 109) consented to participate, and this group did not differ in outcomes compared to those who declined or did not respond to the invitation.

The early language understanding of the study cohort (see Figure 3 for RDLS-III scores at one year post first CI) seems to align with the results of previous studies [1,9,62] indicating that early age at implantation is beneficial for initial linguistic development. We believe that our study protocol can contribute with new knowledge regarding if this relationship between linguistic development and age at implantation continues to be present as the individuals growing up with CIs enter adulthood. The multidisciplinary approach also offers the possibility to study a multitude of other factors that may contribute to linguistic development, beyond age at implantation.

The outcomes of this project will not only provide new insights into the long-term effects of age at first CI but also shed light on the own experiences and perspectives of young people who have grown up with CIs, through a combination of collecting quantitative and qualitative data. There is also potential for conducting interdisciplinary data analyses on topics such as executive functioning, etiology, mental health, HRQoL, and listening in noise among individuals with CIs who received CIs early in life. Such analyses may enhance our current understanding of reciprocal effects and reveal subgroup differences within the cohort, and contribute with new knowledge that may explain some of the yet unknown variability in individuals with CIs.

One limitation is the lack of comprehensive hearing background data in the medical records, particularly regarding age at diagnosis, which was only available for 27 participants (54%). Another limitation of the TAYACI study is that it is not a prospective longitudinal design, and its participants are not fully representative of the broader population of adolescents who use CIs. This is evident in factors such as limited diversity in socioeconomic status (e.g., parental education levels) and the low participation of individuals from multilingual and multicultural backgrounds. Additionally, the study excludes data from individuals with severe and multiple disabilities who presumably were unable to complete the tests used in the sub-studies. A formal sample size calculation was not conducted, as the objective was to recruit the maximum number of participants meeting the inclusion criteria from our center. However, the study includes participants from different regions of the country, ensuring broad geographical representation, as well as individuals with additional diagnoses who were able to complete the assessments and questionnaires. Overall, the cohort captures some characteristics commonly observed in the broader population of individuals with prelingual SNHL or deafness who use CIs. The current cohort reflects characteristics commonly observed in the broader population of individuals with prelingual SNHL or deafness who use CIs.

## 5. Conclusions

Early age at first CI influences early language outcome after one year with CI. The anticipated outcomes of this multidisciplinary study program include an improved understanding of the long-term impacts of early age on CIs in a cohort of young people who were implanted before the age of 30 months. This includes new insights into cognition, language abilities, hearing, vestibular function, etiological factors, mental health, and HRQoL. Furthermore, the TAYACI study will gain insight into the participating adolescent CI users’ own experiences of living with CIs. Importantly, the findings will help identify clinical parameters that can be tailored to maximize benefits for individual CI users, offering critical decision support for clinicians, CI users, and their families in optimizing outcomes.

## Figures and Tables

**Table 1:** Background information of study participants with cochlear implants (*n* = 50).

Variables	*n* (%)
Sex Female Male	27 (54%) 23 (46%)
Etiology Congenital cytomegalovirus infection Connexin 26 Meningitis Hereditary Usher type I Waardenburg’s syndrome X-linked Jervell and Lange-Nielsen syndrome MYO15A Heterozygot för Connexin 26 Mondini dysplasia Pendred’s syndrome Unknown	8 (16%) 6 (12%) 4 (8%) 3 (6%) 3 (6%) 2 (4%) 2 (4%) 3 (6%) 1 (2%) 2 (4%) 1 (2%) 2 (4%) 13 (26%)
Communication mode Spoken Swedish Spoken Swedish (and sign support) Two or more spoken languages Sign language (and spoken Swedish)	42 (84%) 4 (8%) 3 (6%) 1 (2%)
Parents’ age (years) Mothers * Fathers #	Mean 49 (SD: 5.7) min-max: 37–61 Mean 52 (SD: 6.2) min-max: 39–69
Mother’s education level <High school High school Post-secondary education University No response	0 (0%) 7 (14%) 9 (18%) 32 (64%) 2 (4%)
Father’s education level <High school High school Post-secondary education University No response	2 (4%) 7 (14%) 15 (30%) 23 (46%) 3 (6%)
No. of siblings 0 1 2 3 4 or more No response	2 (4%) 23 (46%) 17 (34%) 2 (4%) 4 (8%) 2 (4%)

Notes: etiology status at the time of the study; * missing data = *n* = 3; # missing data = *n* = 5.

**Table 2:** Hearing diagnosis and intervention characteristics (*n* = 50).

Age at Diagnosis of Hearing Loss (Months) (*n* = 27)	Mean (SD) Range
	9.85 (7.47) 1–23
Hearing aids before CI	
Yes	36
No	6
Unknown	8
Age at cochlear implantation (months)	
Age at 1st CI (*n* = 50)	Mean (SD) range
	15.63(6.0) 7.31–29.29
Age at 2nd CI (*n* = 46)	Mean (SD) range
	31 (26.49) 7–149
Type of hearing	
Bilateral implants	46
Unilateral (no contralateral hearing aid)	3
Bimodal (one CI and one hearing aid)	1
CI surgery	
Simultaneous	17
Sequential	32
Unilateral	1
CI brand	
MED-El	46
Cochlear	4
Type of family-centered intervention	
AVT *	28
Karlstadmodellen **	5
Treatment as usual ***	16
No FCEI ****	1

Notes: missing data (*n* = 23). * Auditory Verbal Therapy (AVT) provided at least twice per month over the course of one year by a speech–language pathologist or a teacher of the deaf trained in AVT. ** Structured intervention approach focusing on the network around the child (e.g., for parents and other important caregivers like pre-school teachers) with meetings once a month to evaluate common set goals. *** Unspecified Family-Centered Early Intervention (FCEI) provided on an irregular basis by a teacher of the deaf or a speech–language pathologist. **** No specific support offered for speech and language development.

## Data Availability

The dataset presented in this article is not readily available due to participants’ lack of consent for its dissemination.

## Supplementary Materials

The following supporting information can be downloaded at https://www.mdpi.com/article/10.3390/audiolres15010016/s1. Description of specific materials used in Sub-Studies I–V and references, S1 [8,66,67,68,69,78,79,81,82,83,84,90,92,93,94,95,96,97,98,99,100,101,102,103,104,105,106,107,108,109,110,111,112,113,114,115,116,117,118,119,120,121,122,123,124,125,126,127].
