# Supplementary material for: Teenagers and Young Adults with Cochlear Implants: A Multidisciplinary Follow-Up Study Approach and Baseline Characteristics
Source: Audiol Res. 2025 Feb 12;15(1):16. doi: 10.3390/audiolres15010016 (PMC11851589; doi:10.3390/audiolres15010016)
Supplement: Supplementary file 1 [file audiolres-15-00016-s001.zip › audiolres-3387274-supplementary material.pdf]

S1. Description of specific materials used in Sub-Study I-III and V including references

Sub-Study I: LANGUAGE AND COGNITION

**Ability**	**Reference**
**Figurative language**	
Metaphor comprehension	Norwegian metaphor task [1] translated and adapted in Swedish [2]
**Cognition**	
Non-verbal cognitive ability	Raven's 2 Progressive Matrices [3]
**Executive functions**	
Phonological working memory	Repetition of nonwords and Serial recall of nonwords from SIPS [4]
Phonological working memory (including manipulation)	Reading span task devoloped based on Daneman & Carpenter [5]
Shifting/cognitive flexibility	CTT color trail test [6] or CCTT Children's Color Trail Test [7]
**Reading**	
Reading fluency	Decoding word and decoding nonwords from LS [8]
Reading comprehension	Passage comprehension from Woodcock reading mastery test [9]
**Lexical-semantic abilities**	
Expressive vocabulary	Boston Naming Test [10, 11]
Receptive vocabulary	Peabody Picture Vocabulary Test IV [12]
Word fluency	COWAT (F+A+S), and category verb [13-15]

Questionnaires

**Parental evaluation (participants 18 years and below)**	
Communication (including pragmatics)	CCC-2 [16]
Executive Functions	BRIEF [17]
**Self-evaluation**	
Executive functions	BRIEF [17]

Sub-Study II: HEARING

**Ability or technical assessment**	**Reference and/or test**
**Hearing sensitivity**	
Detection of warble tones in sound field	ISO 8253-2:2009 Acoustics — Audiometric test methods — Part 2: Sound field audiometry with pure-tone and narrow-band test signals
**Spatial hearing**	
Horizontal sound localization accuracy	[18]
Spatial release from masking	[19]
**Recognition of speech in quiet**	
Monosyllabic word recognition	[20]
Speech-in-speech recognition	[21]
**Interaural processing**	
Just noticeable difference of interaural level of a sound	[22]
Just noticeable difference of interaural time of arrival of a sound	[22]
**Spectral discrimination**	
Spectral discrimination	[23]
**Technical integrity of the cochlear implant**	Assessed through commercially available software per cochlear implant manufacturer, e.g. Maestro (version 11.0.2) for Med-El and Custom Sound (version 7.0) for Cochlear.
**Electrically evoked compound action potentials of the auditory nerve**	
Thresholds for Automatically detected neural responses	[24]
**Intracochlear electrode placement**	Photon-counting computer tomography and subsequent image analysis using OTOPLAN software (version 3.1.0) (Med-El, Innsbruck, Austria).

Sub-Study III: VESTIBULAR TESTS AND BALANCE TESTS

**Ability**	**Reference**
**Vestibular functions**	
Semicircular canal's function (gain of eye velocity on head velocity)	Video Head Impulse Test (vHIT) [25]
Sacculus-inferior nerve-cervical pathway function	Air and bone conducted Cervical Vestibular Evoked Myogenic Potentials (cVEMP) – institutional procedure based on:<br>AC stimuli: 500 Hz TB (2-1-2) at 123,5 dB peSPL (insert)<br>BC stimuli: 500 Hz TB (2-1-2) at 134,5 dB peSPL (mastoid)<br>2 trials of 100 sweeps each.
Utriculus-superior nerve-ocular pathway function	Air and bone conducted Ocular Vestibular Evoked Myogenic Potentials (oVEMP) – institutional procedure as upon. [26]
Vestibulo-ocular reflex	Head Impulse Test (HIT) [27]
DVA	Dynamic visual acuity test [28]
**Balance functions**	
Motor Proficiency	Bruininks-Oseretsky Test of Motor Proficiency-Second Edition (BOT-2), Subtest 5: Balance.[29]
Balance and reactions	Kids-Balance Evaluation Systems Test (Kids-BESTest) Section 4: Reactive Postural Responses and Section 5: Sensory Orientation. [30]
Gait and balance	Walking 10 meters with and without head turns (modified from [31])

Questionnaires	***Reference***
**Participant self-evaluation**	
Leisure time physical activity	Saltin-Grimby Physical Activity Level Scale (SGPALS) [32]
Experience of balance, physical activity, participation in physical education in school and motor development	In-house material

Sub-Study V: MENTAL HEALTH and HEALTH-RELATED QUALITY OF LIFE

Ability or area of interest	Reference
**Mental health**	
Self-reported brief behavioral screening (12-17 years)	Strengths and Difficulties Questionnaire (SDQ, ages 4-17) [33], Swedish validation [34]
Parent-reported brief behavioral screening	Strengths and Difficulties Questionnaire (SDQ-P, parent version) [35], Swedish validation [36]
Self-reported brief behavioral screening (18-22 years)	Strengths and Difficulties Questionnaire (SDQ+18, ages 18-22) [37], Swedish validation [38]
Screening for anxiety and/or depressive symptoms	Hospital Anxiety and Depression Scale (HADS) [39], Swedish validation [40]
**Self-efficacy**	
Perceived self-efficacy concerning coping and adaptation in everyday life	General Efficacy Scale (S-GES) [41], Swedish validation [42]
**Everyday life experiences**	
Young people´s everyday life experiences	Young People´s Survey (YPS) [43], Swedish adaption [44]
**Tinnitus**	
Self-report of tinnitus	Tinnitus Functional Index [45], Swedish validation [46]
**General health***	
Screening tool to assess the level of psychological distress and the current state of well-being	General Health Questionnaire (GHQ-12) [47], Swedish validation [48]

Notes: * Young adults only (18-22 years).

References

[1] Kalandadze T, Braeken J, Brynskov C, Næss K-AB. Metaphor Comprehension in Individuals with Autism Spectrum Disorder: Core Language Skills Matter. J Autism Dev Disord 2022;52:316–26. https://doi.org/10.1007/s10803-021-04922-z.

[2] Dahlby Skoog M. The Swedish Metaphor task 2024:320858 *Figshare* 2024. https://doi.org/10.6084/M9.FIGSHARE.26029144.

[3] Raven J., Rust J., Chan F., Zhou X. Raven's 2 progressive matrices, clinical edition (Raven's 2). Pearson.; n.d.

[4] Wass M, Ibertsson T, Lyxell B, Sahlén B, Hällgren M, Larsby B, et al. Cognitive and linguistic skills in Swedish children with cochlear implants – measures of accuracy and latency as indicators of

development. Scandinavian J Psychology 2008;49:559–76. https://doi.org/10.1111/j.1467-9450.2008.00680.x.

[5] Daneman M, Carpenter PA. Individual differences in working memory and reading. Journal of Verbal Learning and Verbal Behavior 1980;19:450–66. https://doi.org/10.1016/S0022-5371(80)90312-6.

[6] Maj M. Evaluation of two new neuropsychological tests designed to minimize cultural bias in the assessment of HIV-1 seropositive persons: A WHO study. Archives of Clinical Neuropsychology 1993;8:123–35. https://doi.org/10.1016/0887-6177(93)90030-5.

[7] Williams J. Children's color trails. Archives of Clinical Neuropsychology 1995;10:211–23. https://doi.org/10.1016/0887-6177(94)00041-N.

[8] Johansson, M-G. Klassdiagnoser i Läsning och skrivning för högstadiet och gymnasiet 2005.

[9] Woodcock, R.W. Woodcock Reading Mastery Tests: Manual. Circle Pines, Minn.: American Guidance Service.; 1973.

[10] Kaplan E, Goodglass H, Weintraub S. Boston Naming Test 2016. https://doi.org/10.1037/t27208-000.

[11] Tallberg IM. The Boston Naming Test in Swedish: Normative data. Brain and Language 2005;94:19–31. https://doi.org/10.1016/j.bandl.2004.11.004.

[12] Dunn, L.M., & Dunn, D.M. Peabody Picture Vocabulary Test, Fourth Edition. Peabody Picture Vocabulary Test, Fourth Edition. Circle Pines, MN: American Guidance Service.; 2007.

[13] Benton, A.L., Hamsher, K. Multilingual Aphasia Examination. Iowa City, IA: AJA Associates; 1989.

[14] Tallberg IM, Ivachova E, Jones Tinghag K, Östberg P. Swedish norms for word fluency tests: FAS, animals and verbs. Scandinavian Journal of Psychology 2008;49:479–85. https://doi.org/10.1111/j.1467-9450.2008.00653.x.

[15] Tallberg IM, Carlsson S, Lieberman M. Children's word fluency strategies: Swedish children's word fluency strategies. Scandinavian Journal of Psychology 2011;52:35–42. https://doi.org/10.1111/j.1467-9450.2010.00842.x.

[16] Bishop, D.V.M. The Children's Communication Checklist, version 2 (CCC-2). London: Pearson.; 2003.

[17] Gioia, G. A., Isquith, P. K., Guy, S. C., & Kenworthy, L. Behavior Rating inventory of Executive Functions. Lutz: PAR.; 2000.

[18] Asp F, Mäki-Torkko E, Karltorp E, Harder H, Hergils L, Eskilsson G, et al. A longitudinal study of the bilateral benefit in children with bilateral cochlear implants. International Journal of Audiology 2015;54:77–88. https://doi.org/10.3109/14992027.2014.973536.

[19] Asp F, Reinfeldt S. Effects of Simulated and Profound Unilateral Sensorineural Hearing Loss on Recognition of Speech in Competing Speech. Ear & Hearing 2020;41:411–9. https://doi.org/10.1097/AUD.0000000000000764.

[20] Lidén G, Fant G. Swedish Word Material for Speech Audiometry and Articulation Tests. Acta Oto-Laryngologica 1954;43:189–204. https://doi.org/10.3109/00016485409130295.

[21] Berninger E, Karlsson KK. Clinical study of Widex Senso on first-time hearing aid users. Scandinavian Audiology 1999;28:117–25. https://doi.org/10.1080/010503999424842.

[22] Eklöf M, Tideholm B. The choice of stimulation strategy affects the ability to detect pure tone inter-aural time differences in children with early bilateral cochlear implantation. Acta Oto-Laryngologica 2018;138:554–61. https://doi.org/10.1080/00016489.2018.1424999.

[23] Stadler S, Leijon A. Prediction of Speech Recognition in Cochlear Implant Users by Adapting Auditory Models to Psychophysical Data. EURASIP J Adv Signal Process 2009;2009:175243. https://doi.org/10.1155/2009/175243.

[24] Van Dijk B, Botros AM, Battmer R-D, Begall K, Dillier N, Hey M, et al. Clinical Results of AutoNRT,™ a Completely Automatic ECAP Recording System for Cochlear Implants. Ear & Hearing 2007;28:558–70. https://doi.org/10.1097/AUD.0b013e31806dc1d1.

[25] Wiener-Vacher SR, Wiener SI. Video Head Impulse Tests with a Remote Camera System: Normative Values of Semicircular Canal Vestibulo-Ocular Reflex Gain in Infants and Children. Front Neurol 2017;8:434. https://doi.org/10.3389/fneur.2017.00434.

[26] Rosengren SM, Colebatch JG, Young AS, Govender S, Welgampola MS. Vestibular evoked myogenic potentials in practice: Methods, pitfalls and clinical applications. Clinical Neurophysiology Practice 2019;4:47–68. https://doi.org/10.1016/j.cnp.2019.01.005.

[27] Cremer P. Semicircular canal plane head impulses detect absent function of individual semicircular canals. Brain 1998;121:699–716. https://doi.org/10.1093/brain/121.4.699.

[28] Rine RM, Braswell J. A clinical test of dynamic visual acuity for children. International Journal of Pediatric Otorhinolaryngology 2003;67:1195–201. https://doi.org/10.1016/j.ijporl.2003.07.004.

[29] Bruininks RH, Bruininks BD. Bruininks-Oseretsky Test of Motor Proficiency, Second Edition 2012. https://doi.org/10.1037/t14991-000.

[30] Dewar R, Claus AP, Tucker K, Ware R, Johnston LM. Reproducibility of the Balance Evaluation Systems Test (BESTest) and the Mini-BESTest in school-aged children. Gait & Posture 2017;55:68–74. https://doi.org/10.1016/j.gaitpost.2017.04.010.

[31] Kollén L, Bjerlemo B, Fagevik Olsén M, Möller C. Static and dynamic balance and well-being after acute unilateral vestibular loss. Audiological Medicine 2008;6:265–70. https://doi.org/10.1080/16513860802504681.

[32] Saltin B, Grimby G. Physiological Analysis of Middle-Aged and Old Former Athletes: Comparison with Still Active Athletes of the Same Ages. Circulation 1968;38:1104–15. https://doi.org/10.1161/01.CIR.38.6.1104.

[33] Goodman R, Meltzer H, Bailey V. The strengths and difficulties questionnaire: A pilot study on the validity of the self-report version. European Child & Adolescent Psychiatry 1998;7:125–30. https://doi.org/10.1007/s007870050057.

[34] Malmberg M, Rydell A, Smedje H. Validity of the Swedish version of the Strengths and Difficulties Questionnaire (SDQ-Swe). Nordic Journal of Psychiatry 2003;57:357–63. https://doi.org/10.1080/08039480310002697.

[35] Goodman R, Ford T, Simmons H, Gatward R, Meltzer H. Using the Strengths and Difficulties Questionnaire (SDQ) to screen for child psychiatric disorders in a community sample. Br J Psychiatry 2000;177:534–9. https://doi.org/10.1192/bjp.177.6.534.

[36] Smedje H, Broman J-E, Hetta J, Von Knorring A-L. Psychometric properties of a Swedish version of the "Strengths and Difficulties Questionnaire." European Child & Adolescent Psychiatry 1999;8:63–70. https://doi.org/10.1007/s007870050086.

[37] Goodman A, Goodman R. Strengths and Difficulties Questionnaire as a Dimensional Measure of Child Mental Health. Journal of the American Academy of Child & Adolescent Psychiatry 2009;48:400–3. https://doi.org/10.1097/CHI.0b013e3181985068.

[38] Persson, A., Löfkvist, U. (6 February 2024) *Swedish back-translation of SDQ+18*. Youth in Mind, sdqinfo.org/py/sdqinfo/b3.py?language=Swedish

[39] Zigmond AS, Snaith RP. The Hospital Anxiety and Depression Scale. Acta Psychiatr Scand 1983;67:361–70. https://doi.org/10.1111/j.1600-0447.1983.tb09716.x.

[40] Jörngården A, Wettergen L, Von Essen L. Measuring health-related quality of life in adolescents and young adults: Swedish normative data for the SF-36 and the HADS, and the influence of age, gender, and method of administration. Health Qual Life Outcomes 2006;4:91. https://doi.org/10.1186/1477-7525-4-91.

[41] Schwarzer R, Jerusalem M. General Self-Efficacy Scale 2012. https://doi.org/10.1037/t00393-000.

[42] Löve J, Moore CD, Hensing G. Validation of the Swedish translation of the general self-efficacy scale. Qual Life Res 2012;21:1249–53. https://doi.org/10.1007/s11136-011-0030-5.

[43] Archbold, S. (2014). *Young People´s Survey (YPS).* [In house material], Department of Clinical Science, Intervention and Technology – CLINTEC, Karolinska Institute

[44] Löfkvist, U. (2014). *Mitt liv med CI: Ungdomsenkäten (Swedish translation of Young People's Survey)* [in house material], Department of Clinical Science, Intervention and Technology – CLINTEC, Karolinska Institute

[45] Meikle MB, Henry JA, Griest SE, Stewart BJ, Abrams HB, McArdle R, et al. The Tinnitus Functional Index: Development of a New Clinical Measure for Chronic, Intrusive Tinnitus. Ear & Hearing 2012;33:153–76. https://doi.org/10.1097/AUD.0b013e31822f67c0.

[46] Hoff M, Kähäri K. A Swedish cross-cultural adaptation and validation of the Tinnitus Functional Index. International Journal of Audiology 2017;56:277–85. https://doi.org/10.1080/14992027.2016.1265154.

[47] Gao F, Luo N, Thumboo J, Fones C, Li S-C, Cheung Y-B. [No title found]. Health Qual Life Outcomes 2004;2:63. https://doi.org/10.1186/1477-7525-2-63.

[48] Lundin A, Åhs J, Åsbring N, Kosidou K, Dal H, Tinghög P, et al. Discriminant validity of the 12-item version of the general health questionnaire in a Swedish case-control study. Nordic Journal of Psychiatry 2017;71:171–9. https://doi.org/10.1080/08039488.2016.1246608.
